# First report of the root parasite *Cansjera rheedei* (Santalales: Opiliaceae) in Taiwan

**DOI:** 10.3897/BDJ.8.e51544

**Published:** 2020-04-10

**Authors:** Po-Hao Chen, An-Ching Chung, Sheng-Zehn Yang

**Affiliations:** 1 Graduate Institute of Bioresources, National Pingtung University of Science and Technology, Neipu Township, Pintung, Taiwan Graduate Institute of Bioresources, National Pingtung University of Science and Technology, Neipu Township Pintung Taiwan; 2 Liouguei Research Center, Taiwan Forest Research Institute, Liouguei District, Kaohsiung, Taiwan Liouguei Research Center, Taiwan Forest Research Institute, Liouguei District Kaohsiung Taiwan; 3 Department of Forestry, National Pingtung University of Science and Technology, Neipu Township, Pintung, Taiwan Department of Forestry, National Pingtung University of Science and Technology, Neipu Township Pintung Taiwan

**Keywords:** *Cansjera rheedei*, newly-recorded genus, *
Opilieae
*, Opiliaceae, Taiwan, taxonomy

## Abstract

**Background:**

The family Opiliaceae in Santalales comprises approximately 38 species within 12 genera distributed worldwide. In Taiwan, only one species of the tribe Champereieae, *Champereia manillana*, has been recorded. Here we report the first record of a second member of Opiliaceae, *Cansjera* in tribe Opilieae, for Taiwan.

**New information:**

The newly-found species, *Cansjera rheedei* J.F. Gmelin (Opiliaceae), is a liana distributed from India and Nepal to southern China and western Malaysia. This is the first record of both the genus *Cansjera* and the tribe Opilieae of Opiliaceae in Taiwan. In this report, we provide a taxonomic description for the species and colour photographs to facilitate identification in the field.

## Introduction

The order Santalales comprises approximately 18 families, 160 genera and more than 2,200 species (Nickrent et al. 2010). The family Opiliaceae in Santalales is divided into four tribes (Agonandreae, Anthoboleae, Champereieae and Opilieae) according to the latest classification, comprising 12 genera and 38 species (Le et al. 2018). The life forms of this family include trees, shrubs and lianas; some species occur in evergreen primary or secondary forest, while others are found in a more seasonal climate. Amongst its genera, *Cansjera* and *Opilia* have been recorded so far from beach forest as root parasites. The morphological characteristics of Opiliaceae are axillary inflorescences of various types, with floral bracts that are often early caducous, the exceptions being in two genera – *Cansjera* and *Melientha* – which have persistent bracts (Le et al. 2018). Members of Opiliaceae are usually monoecious with bisexual flowers, although some dioecious variants have been reported with unisexual flowers. The ovary is superior with a single locule that bears one ovule, developing into a drupe (Nickrent et al. 2010).

The tribes, Champereieae and Opilieae, differ from the other two tribes in the family, Agonandreae and Anthoboleae, by the following characteristics. In Champereieae, the twigs, leaves and pedicels are glabrous; the inflorescence is a panicle; the ovaries are globose to ovoid and the styles are absent. In Opilieae, the twigs, leaves and pedicels are sparsely to densely hairy; the inflorescence is an axillary raceme or spike; ovaries are conical to cylindrical and styles are present (Le et al. 2018).

In Taiwan, only one species of the tribe Champereieae (family Opiliaceae), *Champereia manillana* (Blume) Merr. has been recorded (Yang and Lu 1996). In 2019, an unknown species of Opiliaceae was found in the low-altitude area of Miaoli County in central Taiwan. Based on the information provided by Le et al. (2018), we identified this species as *Cansjera rheedei* J.F. Gmelin, representing the first record of this genus in Taiwan.

## Materials and methods

**Figure 1.**
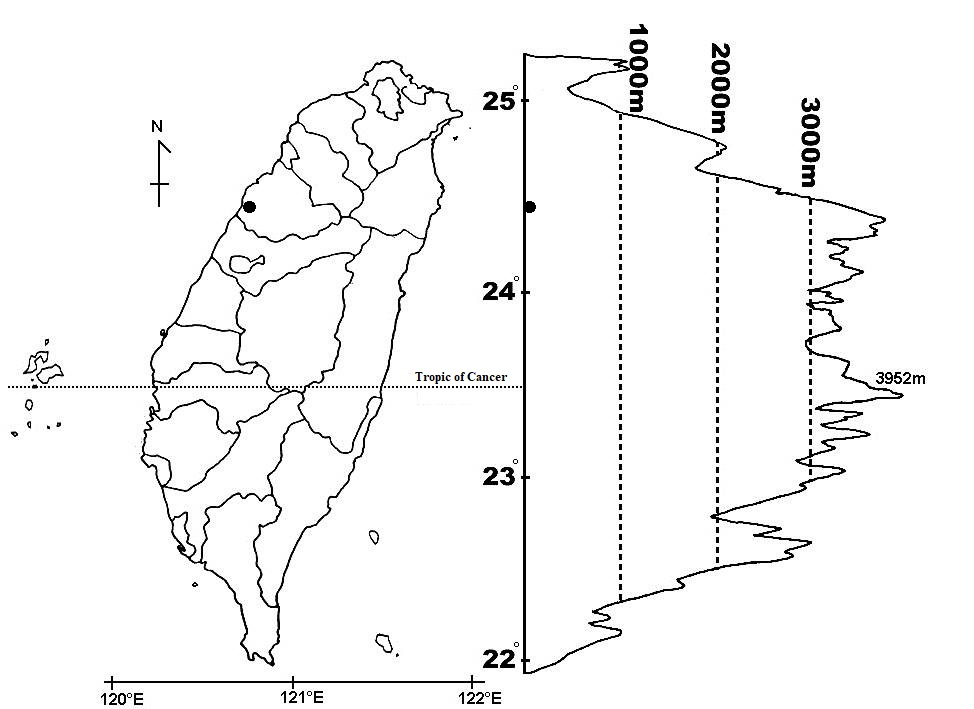
Distribution of *Cansjera rheedei* J.F. Gmelin in Miaoli County, central Taiwan.

All the information on the natural history and species description of *Cansjera rheedei* is based on the plants found in Taiwan. Voucher specimens were deposited in the PPI herbarium at the National Pingtung University of Science and Technology in Pingtung. The distribution map presented in Fig. 1 was based on the information gathered in the field and generated using QGIS vession 3.4 (QGIS Development Team 2018).

## Taxon treatments

### Cansjera rheedei

J. F. Gmelin 1791

98361D5B-E75E-573A-9740-0647633B4A11

urn:lsid:ipni.org:names:607651-1

#### Materials

- **Type status:** Other material. **Occurrence:** recordNumber: P.H. Chen 2583; recordedBy: P.H. Chen and A.C. Chung; **Taxon:** scientificNameID: Cansjera rheedei; **Location:** country: Taiwan; county: Miaoli; locality: Tongxiao Township, Aykoouliau; verbatimElevation: 50-100 m; **Event:** year: 2019; month: 11; day: 24; **Record Level:** type: specimen; collectionCode: PPI
- **Type status:** Other material. **Occurrence:** recordNumber: P.H. Chen 2584; recordedBy: P.H. Chen and A.C. Chung; **Taxon:** scientificNameID: Cansjera rheedei; **Location:** country: Taiwan; county: Miaoli; locality: Tongxiao Township, Aykoouliau; verbatimElevation: 50-100 m; **Event:** year: 2019; month: 11; day: 24; **Record Level:** type: specimen; collectionCode: PPI

#### Description

**Figure 2.**
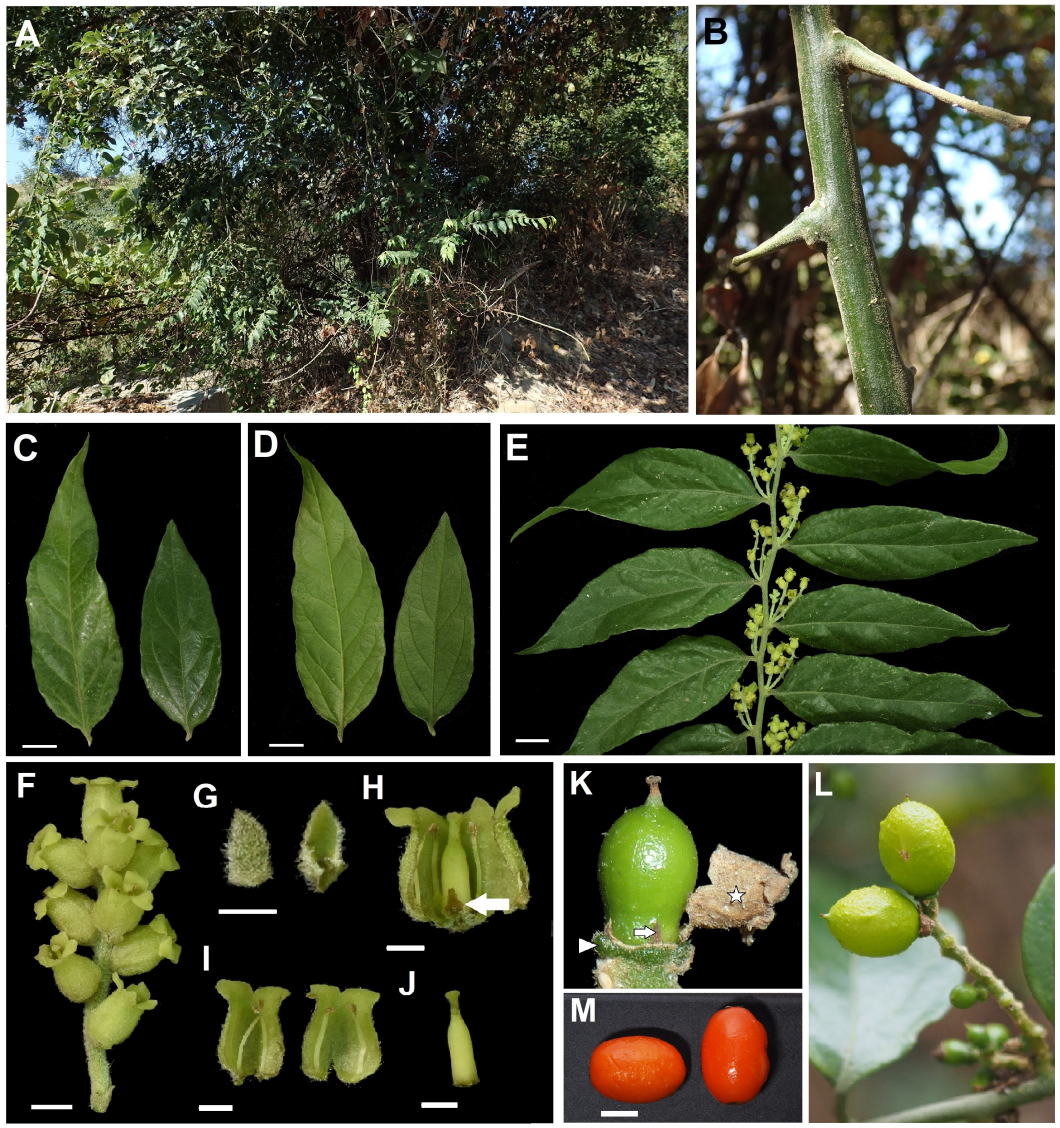
*Cansjera rheedei* J.F. Gmelin. **A.** Habitat; **B.** Thorns derived from short shoots; **C.** Leaf, adaxial view; **D.** Leaf, abaxial view; **E.** Inflorescence, axillary, 1–3 fascicled; **F.** Spikes with 8–13 flowers, perianth urceolate, perianth lobes 4; **G.** Bract, tomentose outside (left) and glossy inside (right); **H.** Dissected flower, with erect scales (arrow); **I.** dissected flower, stamens 4, as many as and opposite to perianth lobes; **J.** Ovary cylindrical; **K.** Immature drupe, disc (arrowhead), scales (arrow) and perianth withered and dropped (star). L. Drupe ellipsoidal; M. Matured drupes, orange to red. Scale bars: C–E = 1 cm; F = 2 mm, G–J = 1 mm, M = 5 mm. All pictures taken by the authors, except K, which was photographed by Hsieh Jo-Ping; L–M, which were photographed by Ming-Hui Chan.

Shrubs or climbing shrubs with spiny stems (Fig. 2A and B), gradually turning into lianas, branchlets densely tomentose. Leaves simple, alternate, lanceolate or ovate, 5.5–9.5 × 2–3 cm, base acute, margin entire or sinuate, apex acuminate, adaxial surface dark green, sparsely pubescent, abaxial surface light green, subglabrous, with obvious reticulate veins, lateral veins 5–7, midrib elevated at both surfaces and densely tomentose, petioles 2–3 mm long, densely tomentose (Fig. 2C-E). Inflorescences spikes, axillary, 1–3 fascicled, 1.5–2 cm long, flowers 8–13; bracts ovate or lanceolate, 1–1.5 × 0.3–0.7 mm, tomentose outside; perianth urceolate, lobes 4, yellowish, tomentose outside, perianth tube 2.5–3 × 2 mm, perianth lobes triangular, 1 × 1 mm, recurved; stamens 4, as many as and opposite to perianth lobes, filaments filiform, 3 mm long, anthers 2-loculed, longitudinal dehiscence; ovary 1, cylindrical, 2 mm long, 1-loculed, ovule 1, style short, stigma capitate, disc 4, inconspicuously lobed, the same number and opposite to the scales; scales 4, erect, the same number and alternate to stamens, triangular, 1 × 1 mm (Fig. 2F-J). Drupe ellipsoidal, orange to red, 11–13 × 8–10 mm, sessile on persistent disc (Fig. 2K-M).

##### Chinese name

山柑藤

#### Distribution

*Cansjera rheedei* is distributed from India and Nepal to southern China and western Malaysia (Hiepko 2008). The population found in Taiwan grows along roadsides at about 50–100 m elevation in the central part of the island (Fig. 1). About forty to fifty mature individuals have been found in the 1.0 ha habitat near a secondary forest with human activities, such as land development and utilisation.

#### Ecology

The population found is located along roadsides where the soil matrix comprises mainly sandy and gravel. Plants grow adjacent to a secondary forest dominated by *Cinnamomum camphora*, whose stem diameter is up to 1 m. Other plants found at the site include *Acacia confusa* Merr. (Fabaceae), *Alpinia zerumbet* (Pers.) B.L. Burtt & R.M. Sm. (Zingiberaceae), *Callerya reticulata* (Benth.) Schot (Fabaceae), *Celtis sinensis* Pers. (Cannabaceae), *Gymnema sylvestre* (Retz.) R. Br. (Apocynaceae), *Ipomoea cairica* (L.) Sweet (Convolvulaceae), *Mallotus japonicus* (Spreng.) Müll. Arg. (Euphorbiaceae), *Mallotus repandus* (Rottler) Müll. Arg., *Panicum maximum* Jacq. (Poaceae), *Trachelospermum jasminoides* (Lindl.) Lem. (Apocynaceae) and *Zanthoxylum nitidum* (Roxb.) DC. (Rutaceae). Recorded flowering and fruiting periods are from November to May.

## Discussion

The discovery of *C. rheedei* in Taiwan represents the first record for both the genus *Cansjera* and the tribe Opilieae in the island (Fig. 2). The individuals we observed are erect shrubs in their earlier stages, gradually turning into lianas that climb over the vegetation, assisted by the thorns present in young branches (Fig. 2A and B). Interestingly, this is the first time this habit has been reported for the species.

Diagnostic characteristics for this newly-recorded species are: leaves sparsely pubescent (Fig. 2C and D), young twigs densely tomentose (Fig. 2E), inflorescences in spikes (Fig. 2F), bisexual flowers with persistent bracts (Fig. 2G and H) and a 4-lobed perianth (Fig. 2I and J), an immature fruit with four fleshy discs, four scales and dried perianth (Fig. 2K), ellipsoidal drupes (Fig. 2L) and mature fruits (Fig. 2 2M). Some differences between the description of *C. rheedei* presented by Hiepko (2008) and the plants found in Taiwan include glabrous leaves (vs. sparsely pubescent in Taiwan), attenuate leaf base (vs. acute), bracts ovate to triangular (vs. ovate or lanceolate), ovary about 1 mm long (vs. 2 mm long) in the former; and the leaf sparsely pubescent, the base acute, the bracts ovate or lanceolate, the ovary being 2 mm long in the latter. Hiepko (2008) referred to the genus *Cansjera* with lobed disc, more or less fleshy scales alternating with stamens and sessible drupe with persistent lacerated perianth. In this study, we found that the perianth gradually wither and blow away, as well as the discs and scales being persistent in the fruit stage (Fig. 2K). To assist with the identification of members of Opiliaceae in Taiwan, we listed the differences between *Cansjera* and *Champereia* in Table 1.

The chemical composition of *Cansjera rheedei* has been investigated by Mounnissamy et al. (2011) and Rama et al. (2018). Phytochemical studies have shown it to demonstrate anti-tumour (Revathi et al. 2018), anti-diabetic and antioxidant activity (Shalini et al. 2010, Ramjith et al. 2013). The phytochemical profile of this newly-recorded species in Taiwan should be compared with *C. rheedei* growing in other countries to determine differences. It is not known if this species was cultivated for medicine in the past. This is worth exploring in more detail.

In China, *C. rheedei* is distributed in Guangdong, Guangxi, Hainan and Yunnan (Qiu and Hiepko 2003). According to this geographical distribution in regions near Taiwan, *C. rheedei* was expected to be found on this island. Only one small population of this species has been found so far in Taiwan and continuous surveys are urgently needed to identify additional sites and understand better its distributional range, abundance and origin.

*Cansjera rheedei*, as with other members of the order Santalales, has been reported as a root parasite (Hiepko 1984, Hiepko and Weber 1978). Similarly, *Cansjera leptostachya* is also a root parasite on Leguminosae and Sapindaceae (Hiepko 1984). In Taiwan, the nearby woody plants and potential hosts of *C. rheedei* include *A. confusa*, *C. sinensis*, *C. camphora* and *M. japonicas*. As the sole population of *C. rheedei*, found to date, is threatened by human activities for land development and utilisation, it is critical to understand the degree of host dependency and affinity to the phylogenetically diverse assemblage of neighbouring trees. This information will prove to be fundamental to design effective long-term management and propagation plans appropriate for a parasitic shrub, including supplementary plantations of suitable hosts near every individual, which might mitigate, to some extent, the impact of habitat disturbance and help preserve this rare species in Taiwan.

## Supplementary Material

XML Treatment for Cansjera rheedei

## Figures and Tables

**Table 1.** Morphological characteristics of *Cansjera* and *Champereia* (Opiliaceae).

**Genus**	**Habit**	**Twigs**	**Flower**	**Bract**	**Perianth tube**	**Reproductive morphology**	**Inflorescence** **type and position**
* Cansjera *	lianas or shrubs	dense with hairs	sessile	small, triangular, persistent	united, urceolate	bisexual	spike, on younger branches
* Champereia *	shrubs or small tree	glabrous	distinct pedicel	none	free, reflexed	polygamous	panicle, on older branches or trunk
